# Comparison of somatic and F+ coliphage enumeration methods with large volume surface water samples

**DOI:** 10.1016/j.jviromet.2018.08.007

**Published:** 2018-08-07

**Authors:** Brian R. McMinn, Eric R. Rhodes, Emma M. Huff, Pauline Wanjugi, Michael M. Ware, Sharon P. Nappier, Mike Cyterski, Orin C. Shanks, Kevin Oshima, Asja Korajkic

**Affiliations:** a United States Environmental Protection Agency, Office of Research and Development, 26 West Martin Luther King Drive, Cincinnati, OH 45268, United States; b Office of Water, 1200 Pennsylvania Avenue NW, Washington, D.C. 20460, United States; c Office of Research and Development, 960 College Station Rd., Athens, GA 30605, United States

**Keywords:** Coliphage, Methods, Ambient water

## Abstract

Coliphages are alternative fecal indicators that may be suitable surrogates for viral pathogens, but majority of standard detection methods utilize insufficient volumes for routine detection in environmental waters. We compared three somatic and F+ coliphage methods based on a paired measurement from 1 L samples collected from the Great Lakes (n = 74). Methods include: 1) dead-end hollow fiber ultrafilter with single agar layer (D-HFUF-SAL); 2) modified SAL (M-SAL); and 3) direct membrane filtration (DMF) technique. Overall, D-HFUF-SAL outperformed other methods as it yielded the lowest frequency of non-detects [(ND); 10.8%] and the highest average concentrations of recovered coliphage for positive samples (2.51 ± 1.02 [standard deviation, SD] log_10_ plaque forming unit/liter (PFU/L) and 0.79 ± 0.71 (SD) log_10_ PFU/L for somatic and F+, respectively). M-SAL yielded 29.7% ND and average concentrations of 2.26 ± 1.15 (SD) log_10_ PFU/L (somatic) and 0.59 ± 0.82 (SD) log_10_ PFU/L (F+ ). DMF performance was inferior to D-HFUF-SAL and M-SAL methods (ND of 65.6%; average somatic coliphage concentration 1.52 ± 1.32 [SD] log10 PFU/L, no F+ detected), indicating this procedure is unsuitable for 1 L surface water sample volumes. This study represents an important step toward the use of a coliphage method for recreational water quality criteria purposes.

Enteric viruses are the leading cause of recreational waterborne disease outbreaks (Sinclair et al., 2009), but detection and enumeration of viral pathogens in environmental waters can be costly, technically challenging, and time consuming. Coliphage may be adequate viral surrogates since they have similar morphological characteristics to enteric pathogenic viruses (King et al., 2011), are present in high levels in wastewater and fecal material (Gantzer et al., 1998; McMinn et al., 2014), exhibit similar reductions to viral pathogens through wastewater treatment processes (Nappier et al., 2006; Pouillot et al., 2015) and replication in the environment is highly unlikely (Jofre, 2009; Muniesa and Jofre, 2004). Somatic and F+ (or male-specific) coliphage enumeration is routinely used by regulatory groups for a variety of applications (e.g. monitoring of groundwater, biosolids, water recycling and aquaculture practices) (Department of Environment and Conservation, 2012; Food and Drug Administration and Interstate Shellfish Sanitation Commission, 2015; North Carolina Environmental Quality, 2011; Queensland Government Environmental Protection Agency, 2005; United States Environmental Protection Agency, 2006). EPA is currently working to develop recreational water criteria for coliphage (United States Environmental Protection Agency, 2015, 2016).

Current coliphage standard culture-based methods include single-agar layer (SAL) plaque assay (American Public Health Association, 2005d; United States Environmental Protection Agency, 2001b), double-agar layer (DAL) plaque assay (American Public Health Association, 2005a,b,c; International Organization for Standardization, 1995, 2000), enrichment (United States Environmental Protection Agency, 2001a) and direct membrane filtration (American Public Health Association, 2005e). These methods recommend test sample volumes ≤ 100 mL, and when applied to surface waters, often result in a high frequency of non-detects (ND) (Abdelzaher et al., 2011; Boehm et al., 2009; Colford et al., 2007; Medema et al., 1995; Viau et al., 2011; von Schirnding et al., 1992; Wade et al., 2010), even when fecal indicator bacteria (*E. coli* or enterococci) are present at 1–2 orders of magnitude higher concentrations (Boehm et al., 2009; Ortega et al., 2009; Viau et al., 2011). A simple solution to decrease the frequency of ND results in contaminated waters is to increase the sample volume tested. However, little is known about the performance of standard coliphage methods with larger surface water sample volumes (≥ 1 L).

In this study, we evaluate the performance of three somatic and F+ coliphage methods with 1 L sample volumes, including dead-end hollow fiber ultrafiltration (Mull and Hill, 2012; Smith and Hill, 2009) with SAL (D-HFUF-SAL) (McMinn et al., 2017), an improved direct membrane filtration procedure (DMF) (Sobsey et al., 2004) and SAL (United States Environmental Protection Agency, 2001b) modified to accommodate a 1 L sample volume (M-SAL). Performance comparisons are based on paired measurements from a series of 1 L surface water samples collected from the Great Lakes region. In addition, practical implementation factors such as the occurrence of ND results, cost, and sample processing time are discussed.

Surface waters originated from Lake Michigan (Washington Park Beach in Michigan City, Indiana) (n = 37) and nearby Trail Creek (n = 37). Samples were collected during the 2015 Great Lakes beach season at a frequency of five samples per week. The sample collection procedures are detailed elsewhere (Wanjugi et al., 2018). The D-HFUF-SAL (McMinn et al., 2017) and DMF (Sobsey et al., 2004) methods were performed as previously described, while media and reagents were in-creased 10-fold for the M-SAL procedure (United States Environmental Protection Agency, 2001b); please see supplemental information for more details.

All data were log10 transformed and expressed as plaque forming unit (PFU) per liter (L) for positive samples only as ND samples were not included in the concentration data. Statistical analyses were performed using SigmaPlot version 13.0 (Systat Software, inc., San Jose, CA). One-way analysis of variance (ANOVA) followed by Holm-Sidak multiple comparisons or Kruskal-Wallis ANOVA on ranks (followed by Tukey tests) were applied to somatic and F+ coliphage datasets (from both sites), while Wilcoxon signed rank tests were used for overall comparisons between the coliphage types or sites.

Method performance metrics are summarized in Table 1, while coliphage concentrations are depicted in Fig. 1. In Lake Michigan samples, somatic coliphage were detected more frequently and at higher levels using the D-HFUF-SAL compared to the other two methods tested (Table 1, Fig. 1A). Average concentrations for positive samples en-umerated using D-HFUF-SAL method (1.65 ± 0.63 [SD] log_10_ PFU/L) were comparable to M-SAL (*p* = 0.124, 1.26 ± 0.67 [SD] log_10_ PFU/L), but concentrations obtained by DMF (0.30 ± 0.44 [SD] log_10_ PFU/L) (Fig. 1A) were significantly (*p* < 0.001) lower than either D-HFUF-SAL or M-SAL methods. The DMF method did not yield any F+ coliphage results from Lake Michigan samples, but they were detected using the other two methods (Fig. 1A) with D-HFUF-SAL (0.32 ± 0.34 [SD] log10 PFU/L) method resulting in higher average concentration for positive samples as compared to the M-SAL (0.05 ± 0.26 [SD] log_10_ PFU/L) (Fig. 1A). D-HFUF-SAL yielded significantly higher concentrations of F+ coliphage (*p* < 0.001) for positive samples than either M-SAL or DMF methods, but there was no statistically significant difference in F+ coliphage concentrations obtained by the M-SAL and DMF methods (*p* = 0.899).

Somatic coliphage were consistently detected in Trail Creek water samples irrespective of the method (Table 1). D-HFUF-SAL and M-SAL exhibited similar somatic coliphage mean concentrations for positive samples in Trail Creek samples; 3.38 ± 0.43 (SD) log_10_ PFU/L and 3.25 ± 0.44 (SD) log_10_ PFU/L, respectively (*p* = 0.252) (Fig. 1B). Significantly lower levels (*p* < 0.001) of somatic coliphage for positive samples were observed with the DMF method (2.75 ± 0.49 [SD] log_10_ PFU/L) (Fig. 1B). Comparable (*p* = 0.659) average concentrations of F+ coliphage in positive samples were found in Trail Creek waters using the D-HFUF-SAL method (1.25 ± 0.68 [SD] log_10_ PFU/L) and M-SAL (1.22 ± 0.85 [SD] log_10_ PFU/L), but concentrations obtained by the DMF method were significantly lower (*p* < 0.001) (Fig. 1B). Overall, somatic coliphage concentrations were significantly higher than the F+ coliphage, irrespective of method and matrix (*p* < 0.001), and concentrations of both coliphage types were typically higher in the Trail Creek samples compared to the Lake Michigan samples (*p* < 0.001).

Regarding frequency of NDs, D-HFUF-SAL exhibited the lowest ND range overall, with 0%–2.7% (somatic) and 5.4%–35% (F+) (Table 1). M-SAL resulted in the second lowest ND occurrence (0%–8.1% for somatic; 16.2%–94.6% for F+), followed by DMF (0%–62.2% for somatic and 100% for F+) (Table 1).

When considering cost associated with each method, the assessment is based on current manufacturers prices for disposable items such as petri dishes and filters, as well as various chemicals and reagents (e.g. agar, tryptic soy broth, nalidix acid, streptomycin, ampicillin, X-gal, IPTG, MgCl_2_, CaCl_2_, MgSO_4_, tryptone, glucose, Tween 80, Antifoam Y-30, sodium hexametaphosphate) but we’ve excluded common laboratory disposables such as serological pipettes, pipette tips, gloves and similar. Regarding the processing time, we have assumed that sample is being processed by a single analyst familiar with the routine water quality assessment (e.g. membrane filtration and /or defined substrate technology for enumeration of *E. coli* and enterococci) and possessing basic knowledge of microbiological culture techniques. The cost per sample for D-HFUF-SAL and DMF was comparable, while M-SAL was approximately ten times higher, reflecting the increase in supplies and reagents needed to process 1 L sample volumes (Table 2). D-HFUF-SAL required the least amount of time to process a single sample, followed by M-SAL. The time required for DMF varied greatly due to the potential for substantial membrane clogging (Table 2).

Overall, the D-HFUF-SAL method yielded the highest levels of both coliphage types and the lowest incidence of ND results, suggesting that the addition of an ultrafiltration step to the SAL procedure enhances method performance for surface water applications. These results are similar to a recent study, where a low percentage of ND results was observed in surface waters ranging from 0% (somatic) to 25% (F+) (McMinn et al., 2017). M-SAL method performed similarly to D-HFUF-SAL when incidence of ND samples was low (i.e. < 10%), but it yielded significantly lower coliphage concentrations when incidence of NDs was high (e.g. F+ coliphage in Lake Michigan samples). However, the high cost per sample and extended sample processing time may render this approach unfeasible for sample volumes greater than 100 mL. While an earlier study utilizing DMF reported detectable somatic and F+ coliphage in 88% and 48% of estuary samples, respectively (Love et al., 2010), a much lower incidence was observed with Great Lake region surface water samples tested in this study. It is challenging to compare study outcomes due to different water sample types (estuary versus river/beach samples), as well as potentially different levels and sources of fecal contamination. However, it is worth noting that the two studies utilized different filtration strategies where 100 mL samples were divided into 10 separate subsamples in one approach (Love et al., 2010), and a 1 L volume was passed through a single filter when possible in this study as recommended by the method developers (Sobsey et al., 2004). This practice often led to membrane clogging, longer filtration times, and in many cases, resulted in a layer of particulate matter on the filter surface, which may have interfered with plaque formation and visualization (typically when turbidity exceeded 10 NTU). A different tactic, where a 1L sample is subdivided into 10 × 100mL subsamples, may have yielded a different result, but would undoubtedly be more expensive and time consuming. In summary, we compared three (D-HFUF-SAL, M-SAL, DMF) somatic and F+ coliphage methods on paired 1 L samples collected from the Great Lakes region. Our results suggest that D-HFUF-SAL significantly outperformed the other two methods as it provided the lowest frequency of non-detects and the highest concentration of coliphages. This study represents an important step toward the use of a coliphage method for surface waters.

## Supplementary Material

Supp Data

## Figures and Tables

**Fig. 1.**
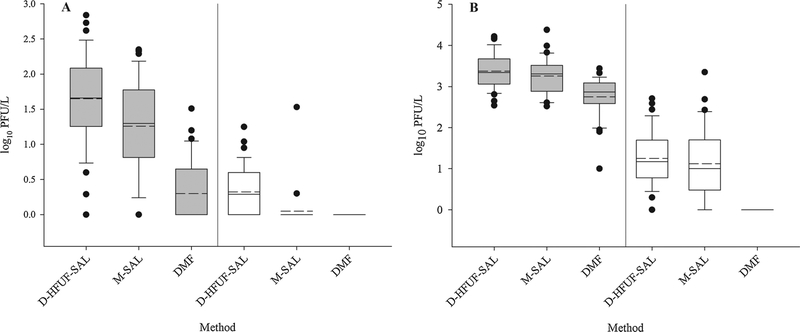
Concentrations of somatic (shaded boxes) and F+ (empty boxes) coliphage in positive samples for Lake Michigan Beach (panel A) and Trail Creek (panel B) samples. Boxes are delimited by 25th and 75th percentiles, solid line within the box represents median and dashed line represents average. Whiskers are 10th and 90th percentile values. Values outside of the range are depicted as black dots. Dead-end hollow fiber ultrafiltration with single agar layer (D-HFUF-SAL), modified single agar layer (M-SAL), direct membrane filtration (DMF). N = 37 at each sample location for each coliphage type and method.

**Table 1:** Performance metrics of D-HFUF-SAL, M-SAL and DMF in Lake Michigan and Trail Creek samples.

Coliphage type	Lake Michigan			Trail Creek		
	D-HFUF-SAL^a^	M-SAL^a^	DMF^a^	D-HFUF-SAL	M-SAL	DMF

Somatic	2.7%	8.1%	62.2%	0%	0%	0%
F+	35.1%	94.6%	100%	5.4%	16.2%	100%

a Dead-end hollow fiber ultrafiltration with single agar layer plaque assay (D-HFUF-SAL), modified single agar layer plaque assay (M-SAL), direct membrane filtration (DMF).

**Table 2:** Cost and time requirements per sample for D-HFUF-SAL, M-SAL and DMF methods.

Logistics	Method^a^		
	D-HFUF-SAL	M-SAL	DMF

Cost per sample^b^	$30–$40	$250–$300	$30–$40
Time required^c^	10–15 min	25–30 min	5–60 min^d^

a Dead-end hollow fiber ultrafiltration with single agar layer plaque assay (D-HFUF-SAL), modified single agar layer plaque assay (M-SAL), direct membrane filtration (DMF).

b Based on the current manufacturer pricing for disposable items such as petri dishes and filters, as well as various chemicals and reagents (e.g. agar, tryptic soy broth, nalidix acid, streptomycin, ampicillin, X-gal, IPTG, MgCl_2_, CaCl_2_, MgSO4, tryptone, glucose, Tween 80, Antifoam Y-30, sodium hexametaphosphate).

c Assuming single analyst familiar with the routine water quality assessment procedures and possessing basic knowledge of microbiological culture techniques is processing the sample.

d Processing time was highly variable depending on number of filters required and filtering speed.
