# Supplementary material for: Comparison of somatic and F+ coliphage enumeration methods with large volume surface water samples
Source: J Virol Methods. Author manuscript; Available in PMC 2020 Mar 20. (PMC7082814; doi:10.1016/j.jviromet.2018.08.007)
Supplement: Supp Data [file NIHMS1051353-supplement-Supp_Data.docx]

**Supplemental information for: Comparison of somatic and F+ coliphage enumeration methods with large volume surface water samples**

Brian R. McMinn^a^, Eric R. Rhodes^a^, Emma M. Huff^a^, Pauline Wanjugi^a,d^, Michael M. Ware^a^, Sharon P. Nappier^b^, Mike Cyterski^b^, Orin C. Shanks^a^, Kevin Oshima^a^ and Asja Korajkic^a#^

United States Environmental Protection Agency

Office of Research and Development^a^

26 West Martin Luther King Drive, Cincinnati, OH 45268

Office of Water^b^

1200 Pennsylvania Avenue NW, Washington, D.C. 20460

Office of Research and Development^c^

960 College Station Rd., Athens, GA, 30605

*New York State Department of Health^d^

Wadsworth Center

120 New Scotland Avenue, Albany, New York 12208

# corresponding author

*present address

E-mail: korajkic.asja@epa.gov

The D-HFUF-SAL method was performed as previously described (McMinn et al., 2017). Briefly, 2L of water was passed through a single-use 15S Asahi Kasei Rexeed ultrafilter (Dial Medical Supply, Chester Springs, PA) using a peristaltic pump. Filter was eluted by passing ~200 mL of elution solution (0.01% Tween 80, 0.01% sodium hexametaphosphate, 0.001% Antifoam Y-20 [Sigma-Aldrich, St. Louis, MO]) in the clockwise, counter-clockwise and finally clockwise direction for 1 minute each. The eluate (~ 200 mL) was divided into two even portions and processed using the SAL procedure, as previously described (United States Environmental Protection Agency, 2001). Briefly, 100 mL elution solution (per coliphage type) was mixed with 100 mL of molten media (2X tryptic soy agar [Fisher Scientific, Waltham, MA]) along with the 10 mL of appropriate bacterial host (*E. coli* CN-13 [ATCC 700609] for somatic and *E. coli* Famp [ATCC#700891] for F+ coliphage) in the mid-log growth phase, 2 mL of appropriate antibiotic solution (100 μg/ mL nalidixic acid for CN-13 and 15 μg/ mL streptomycin/ampicillin for Famp [ Fisher Scientific, Waltham, MA]) and 4 M MgCl_2_ (Sigma-Aldrich, St. Louis, MO). Contents were inverted several times to mix, plated on five large petri dishes (150 mm diameter) and incubated at 37°C for 16-18 hours. Previously published protocols (Sobsey et al., 1990; Sobsey et al., 2004) were followed for the DMF method. Briefly, tryptone-glucose-NaCl-Tween bottom agar plates and top agar overlay (Sobsey et al., 1990) (prepared fresh each day samples were analyzed) were amended with X-Gal (5-bromo-4-chloro-3-indolyl-beta-D- galactopyranoside, [Sigma-Aldrich, St. Louis, MO]), IPTG (isopropyl-beta-D-thiogalactopyranoside, [Sigma-Aldrich, St. Louis, MO]) (Sobsey et al., 2004) and appropriate antibiotics (as described above for D-HFUF-SAL method). Approximately, 2-3 mL (per sample) of top agar was amended with 100 µl of appropriate bacterial host in mid-log growth phase (as described above for the D-HFUF-SAL method). Top agar was poured on the bottom agar plates and allowed to solidify. A total of 1L sample volume was amended with 5M MgCl_2_ (Sobsey et al., 2004) and passed through one or more membrane filters (0.45 µm pore size, 47 mm diameter) as multiple filters were required in some instances due to clogging. Following filtration, membranes were placed (gridded-side down) on bottom agar plates containing top agar and were incubated for 16-18 hours at 37°C. The SAL method was originally described in USEPA Method 1602 for analysis of 100 mL volumes of groundwater. The procedure (M-SAL) was performed as described above for D-HFUF-SAL, with the following modification: bacterial hosts, media and reagents were increased 10-fold to accommodate 1L sample volumes. During each sampling event, a positive control and two negative controls were performed for each method. Positive control consisted of adding either Phi X174 (somatic coliphage, ATCC#13706-B1) or MS2 (F+ coliphage, ATCC# 15597-B1) to 100 mL of sterile 0.01 M phosphate buffer solution (PBS) pH 7.4 (Sigma-Aldrich, St. Louis, MO), followed by sample processing as described above. Negative controls consisted of method blanks where sample was substituted with 0.01M PBS and media sterility checks where plates containing only agar were incubated. For the duration of the study, positive controls yielded expected results (i.e. plaques characteristic of each coliphage type) and no plaques were observed on any of the negative controls indicating absence of contamination.

**References**

McMinn, B.R., Huff, E.M., Rhodes, E.R. and Korajkic, A., 2017. Concentration and quantification of somatic and F+ coliphages from recreational waters. Journal of virological methods 249, 58-65.

Sobsey, M.D., Schwab, K.J. and Handzel, T.R., 1990. A Simple Membrane-Filter Method to Concentrate and Enumerate Male-Specific Rna Coliphages. J Am Water Works Ass 82, 52-59.

Sobsey, M.D., Yates, M.V., Hsu, F.C., Lovelace, G., Battigelli, D., Margolin, A., Pillai, S.D. and Nwachuku, N., 2004. Development and evaluation of methods to detect coliphages in large volumes of water. Water Sci Technol 50, 211-217.

United States Environmental Protection Agency. 2001. Method 1602: Male-specific (F+) and Somatic Coliphage in Water by Single Agar Layer (SAL) Procedure, Washington, D.C.
